# Broadband and High Sensitive Time-of-Flight Diffraction Ultrasonic Transducers Based on PMNT/Epoxy 1–3 Piezoelectric Composite

**DOI:** 10.3390/s150306807

**Published:** 2015-03-19

**Authors:** Dongxu Liu, Qingwen Yue, Ji Deng, Di Lin, Xiaobing Li, Wenning Di, Xi’an Wang, Xiangyong Zhao, Haosu Luo

**Affiliations:** 1 Key Laboratory of Inorganic Functional Materials and Devices, Shanghai Institute of Ceramics, University of Chinese Academy of Sciences, 215 Chengbei Road, Jiading, Shanghai 201800, China; E-Mails: liudxu@outlook.com (D.L.); yue.qingwen@student.sic.ac.cn (Q.Y.); dengjie@student.sic.ac.cn (J.D.); nick_lindi@163.com (D.L.); lxbing@mail.sic.ac.cn (X.L.); dwn@mail.sic.ac.cn (W.D.); wang1972wang@hotmail.com (X.W.); xyzhao@mail.sic.ac.cn (X.Z.); 2 University of Chinese Academy of Sciences, Beijing 100049, China

**Keywords:** time-of-flight diffraction, ultrasonic transducer, PMNT, 1–3 piezoelectric composite

## Abstract

5–6 MHz PMNT/epoxy 1–3 composites were prepared by a modified dice-and-fill method. They exhibit excellent properties for ultrasonic transducer applications, such as ultrahigh thickness electromechanical coupling coefficient *k*_t_ (85.7%), large piezoelectric coefficient *d*_33_ (1209 pC/N), and relatively low acoustic impedance *Z* (1.82 × 10^7^ kg/(m^2^·s)). Besides, two types of Time-of-Flight Diffraction (TOFD) ultrasonic transducers have been designed, fabricated, and characterized, which have different matching layer schemes with the acoustic impedance of 4.8 and 5.7 × 10^6^ kg/(m^2^·s), respectively. In the detection on a backwall of 12.7 mm polystyrene, the former exhibits higher detectivity, the relative pulse-echo sensitivity and −6 dB relative bandwidth are −21.93 dB and 102.7%, respectively, while the later exhibits broader bandwidth, the relative pulse-echo sensitivity and −6 dB relative bandwidth are −24.08 dB and 117.3%, respectively. These TOFD ultrasonic transducers based on PMNT/epoxy 1–3 composite exhibit considerably improved performance over the commercial PZT/epoxy 1–3 composite TOFD ultrasonic transducer.

## 1. Introduction

Time-of-flight diffraction (TOFD) is a high accuracy detection technology based on the weak diffraction signals of ultrasonic waves from defects for the non-destructive testing (NDT) applications. The broadband and high sensitive ultrasonic transducers play a key role in the TOFD detection system. Lead zirconate titanate (PZT) ceramics, since the discovery in the 1950s, have been widely used in the fabrication of ultrasonic transducers for NDT applications due to their high electromechanical properties (e.g., *d*_33_ = 700 pC/N, *k*_33_ = 0.70, *etc*.) and essentially mature fabrication processes. In order to further improve the performances, PZT/epoxy 1–3 composites with high electromechanical coupling factor *k*_t_ (0.59) and low acoustic impedance Z (1.34 × 10^7^ kg/(m^2^·s)) were also applied in many kinds of transducers, which exhibit improved sensitivity and broad bandwidth [1].

Recently, relaxor ferroelectric single crystals Pb(Mg_1/3_Nb_2/3_)O_3_−xPbTiO_3_ (PMNT) have been grown and studied a lot because of their ultrahigh piezoelectric, dielectric and electromechanical properties near the morphotropic phase boundary (MPB). The piezoelectric coefficient *d*_33_, longitudinal electromechanical coupling factor *k*_33_ and thickness-mode electromechanical coupling factor *k*_t_ can reach up to 2000 pC/N, 0.90 and 0.62, respectively, for [001] oriented PMNT single crystals [2,3,4,5,6]. Using a 1–3 connectivity, the behavior of the PMNT single crystal can be further improved for ultrasonic transducer applications [7,8]. First, the structure of the 1–3 composite reduces the lateral vibration mode and utilizes a high longitudinal coupling coefficient *k*_33_ (90%) instead of a low thickness coupling coefficient *k*_t_ (59%), leading to much efficient conversion between electrical and mechanical energy as compared to the single phase material. Second, the structure of the 1–3 composite would lower the acoustic impedance *Z* and mechanical quality factor *Q*_m_ (the reciprocal of mechanical loss), being beneficial in high damping, broadband transducers design. Third, the dielectric property of the 1–3 composite can be easily tuned by varying the active phase volume fraction. Consequently, the electrical properties of the transducer using this kind of composites can be easily tailored to match the requirements of driving and receiving electronics.

PMNT single crystals and their composites have been applied in the field of NDT over the past several years, such as dual element transducer and angle beam transducer [8,9,10,11,12], while they were limited to some low-frequency range of NDT applications. In this work, 5–6 MHz PMNT/epoxy 1–3 composites were prepared by a modified dice-and-fill technology. Based on the prepared piezoelectric composites, two types of TOFD ultrasonic transducers with different matching layer schemes were designed, fabricated, the performances in the detection of a backwall of 12.7 mm polystyrene were characterized and compared to commercial PZT/epoxy 1–3 composite TOFD ultrasonic transducer.

## 2. Composite Preparation

High-quality PMNT single crystal with rhombohedral phase composition was grown directly from the melt by the modified Bridgman method [13,14]. The single crystal was oriented along the [001] direction and then cut into 15 × 15 × 0.8 mm wafers. The wafers were diced along two perpendicular directions using a Disco DAD 320 automatic dicing saw (Disco, Tokyo, Japan). Since the thickness of the 5 MHz composite is only about 0.2mm, and considering the PMNT volume fraction and height to width ratio of piezoelectric pillars, the dicing pitch and depth were set as 0.1 mm and 0.35 mm, respectively, and a 24-µm-thick nickel/diamond blade was adopted. Besides, the suitable feed speed (1~2 mm/s) and water flow (0.6~0.8 L/min) are also important for the dicing quality. After dicing, a low-viscosity epoxy Epo-Tek 301 (Epoxy Technology Inc., Billerica, MA, USA) was filled into kerfs and vacuumed to remove the trapped bubbles, then cured at 60 °C for more than 3 h. Subsequently, the polymers and supernumerary single crystal were lapped away from the top and bottom sides of the composite. The final thickness of the composite was reduced to 0.2 mm for resonating at around 5 MHz, then Cr/Au electrodes were sputtered on the two main faces for 2 min and 7 min with the rate of ~15 nm/min and 30 nm/min, respectively. Finally, the samples were poled under an electric field of 1 kV/mm in the air at 80 °C for 30 min.

**Figure 1:**
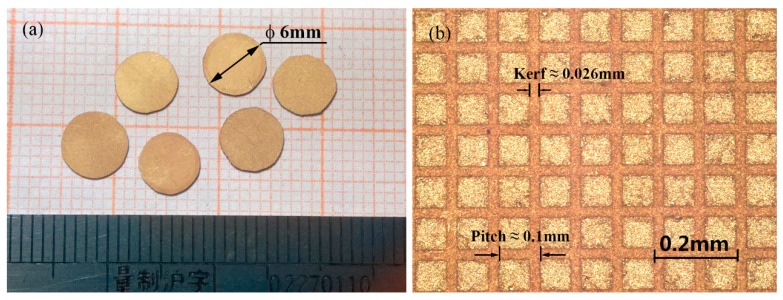
(**a**) Photograph of the prepared PMNT/epoxy 1–3 composites; (**b**) Enlarged image of a randomly selected area on the composite.

**Figure 2:**
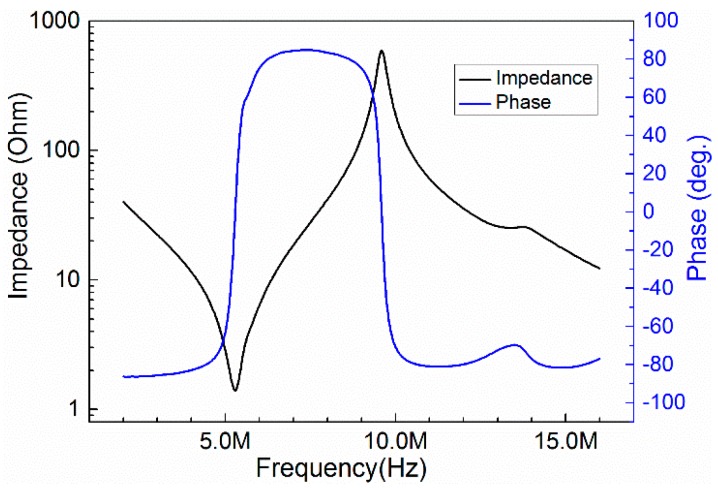
The impedance and phase angle spectra of the prepared PMNT/epoxy 1–3 composite.

Figure 1 show the as-prepared composites and the micrograph of the sample, respectively. There were no cracks and the crystal rods stood well in epoxy matrix. Since the measured width of the dicing kerfs was 0.026 mm, the practical volume fraction of PMNT single crystal was calculated to be 54.8%. Here, the piezoelectric coefficients *d*_33_ was measured by a quasistatic Berlincourt meter at about 55 Hz, and the density ρ was determined by the Archimedes principle. Figure 2 shows the impedance and phase angle spectra of the composite measured by an Agilent impedance analyzer 4294A (Agilent Technologies, Santa Clara, CA, USA), and the phase angle achieved 85.2 degrees indicated that the degree of polarization is relatively complete. The following parameters were calculated according to the IEEE standards on Piezoelectricity:

Electromechanical coupling factor *k*_t_:

(1) $${k_{t}}^{2}=\frac{\pi }{2}\cdot \frac{f_{P}}{f_{s}}\tan\left(\frac{\pi }{2}\cdot \frac{f_{p}-f_{s}}{f_{s}}\right)$$

where *f*_s_ and *f*_p_ represent the maximum conductance frequency and maximum resistance frequency, respectively.

Sound velocity $\;v_{l}\;$ and acoustic impedance Z:

(2) $$v_{l}=2f_{p}\cdot t$$

(3) $$\text{Z}=v_{l}\cdot \rho$$

where *t* is the thickness of the sample.

Mechanical quality factor *Q*_m_:

(4) $$Q_{m}=\frac{f_{s}}{f_{1}-f_{2}}$$

where *f*_1_ and *f*_2_ are frequencies at 3 dB down the maximum admittances.

The measured and calculated electric and acoustic properties of the prepared composite and those of traditional piezoelectric materials are shown in Table 1. The results indicate that the properties of PMNT/epoxy 1–3 composite are obviously superior to those of commercial PZT ceramic and their 1–3 composite. Meanwhile, the 1–3 composite exhibits several advantages over the single phase material, such as much higher thickness electromechanical coupling coefficient, relatively lower acoustic impedance and mechanical quality factor. Thus, PMNT/epoxy 1–3 composite is very beneficial to the preparation of high sensitivity and broad bandwidth TOFD transducers.

**Table 1:** The properties of the prepared PMNT/epoxy 1–3 composite and some other common piezoelectric materials.

	*ρ* (kg/m^3^)	$\varepsilon_{T}^{33}$ @ 1 kHz	*d*_33_ (pC/N)	*k*_t_	*Q*_m_	Dielectric Loss @ 1 kHz (tan δ)	*N_t_* (Hz·m)	*Z* ($\times 10^{6}\;$ kg/(m^2^·s))
PMNT	8100	5500	2000	0.62	100	0.005	2300	37
PMNT/epoxy 1–3 composite	4955	2134	1208	0.857	15	0.012	1830	18.2
PZT-5H	7500	3100	600	0.51	65	0.018	1950	34
PZT/epoxy 1–3 composite	4190	3400	593	0.59	20	0.024	1600	13.4

## 3. TOFD Transducer Design and Fabrication

### 3.1. Design and Simulation

A transducer rings at its natural frequency once it is excited by an electrical source. Since the piezoelectric material itself exhibits much higher acoustic impedance than that of the common acoustic loads, such as human tissues for biomedical ultrasound applications and the wedge or the delay line for industrial non-destructive detections, a substantial part of the acoustic energy would be lost at the rear interface and not directed into the forward direction, resulting in poor resolution and sensitivity, if not properly matched acoustically.

The matching layer is known as acoustic transformer between the piezoelectric material and the load material, which can improve the transducer performance significantly. According to the KLM model [15], the matching layer thickness approaches $\lambda_{m}/4\;$ and acoustic impedance of the matching layer material $Z_{m}$ is:

(5) $$Z_{m}=\sqrt[{3}]{Z_{p}\cdot {Z_{l}}^{2}}$$

where $\lambda_{m}$ is the wavelength in the matching layer material, $Z_{p}$ and $Z_{l}$ are the acoustic impedances of piezoelectric material and the load material, respectively [16]. For the TOFD ultrasonic transducer, the piezoelectric material is PMNT/epoxy 1–3 composite (1.82 $\;\times 10^{7}\;$ kg/(m^2^·s)), and the wedge material is polystyrene (2.48 $\;\times 10^{6\;}$ kg/(m^2^·s)), the acoustic impedances of the matching layer was calculated to be 4.82 $\;\times 10^{6}\;$ kg/(m^2^·s)using formula 5. Sometimes, a larger acoustic impedance of matching layer scheme was also adopted to obtain higher sensitivity according to the simulation and previous experiments. Here, in order to satisfy different performance requirements of the TOFD ultrasonic transducers for NDT applications, two matching layer schemes with the acoustic impedance of 4.8 $\;\times 10^{6}\;$ kg/(m^2^·s) and 5.7 $\;\times 10^{6}$ kg/(m^2^·s), respectively, were applied to the design of TOFD transducers.

The backing layer is another important component in a piezoelectric ultrasonic transducer. In fact, 100% transmission is impossible for only considering the matching layer. Due to the acoustic mismatch between the air and the piezoelectric material, the reflected wave reverberates inside the transducer element. This would cause long ring-down of the ultrasonic pulse, which is the so-called ringing effect. Therefore, the fabrication of backing layer is very necessary to avoid this ringing effect and improve the quality of echo signal. The backing layer is usually a highly attenuative, high density material that is used to control the vibration of the transducer by absorbing the energy radiating from the back face of the active element. Taking into account of the sensitivity and bandwidth, a backing layer with acoustic impedance of about 8.5 $\;\times 10^{6}\;$ kg/(m^2^·s) was adopted.

In this work, a low-viscosity epoxy Epo-Tek 301 (Epoxy Technology Inc.) mixed with zirconia or tungsten powder were selected as the matching layer and backing layer materials, and the properties of front matching and backing layers used for the TOFD transducer fabrication are listed in Table 2.

**Figure 3:**
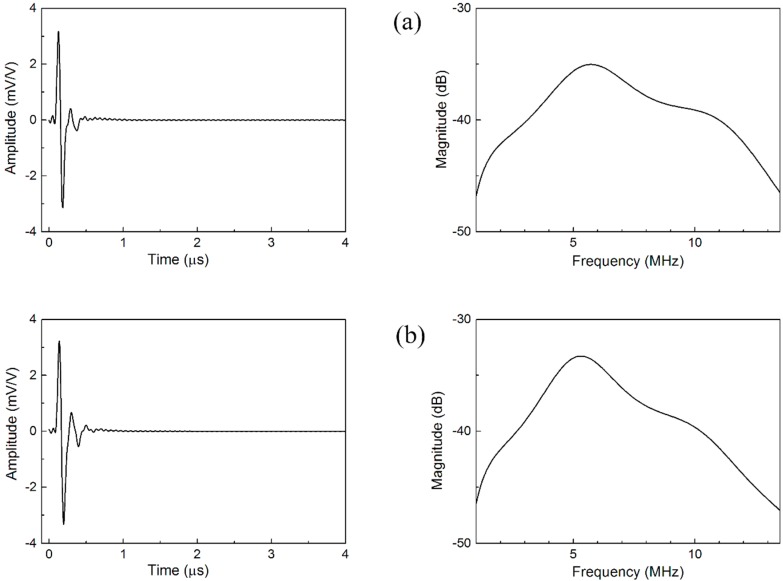
The simulated waveforms and frequency spectra of the designed TOFD ultrasonic transducers with (**a**) scheme I; (**b**) scheme II.

The performance of the ultrasonic transducer can be predicted using the existing one-dimensional circuit KLM model (PiezoCAD, Sonic Concepts, Inc., Washington, WA, USA). From the PiezoCAD simulation, center frequency (*f*_c_), −6 dB bandwidth (*BW* @ −6 dB), relative pulse-echo sensitivity and the pulse width (*PW*) were analyzed. The simulated waveforms and frequency spectra and performance parameters of the designed TOFD ultrasonic transducers with two matching layer schemes are extracted from Figure 3 and listed in Table 3.

**Table 2:** The properties of the passive materials used for the TOFD transducer fabrication.

Material	Use		Weight Ratio (Epoxy:Powder)	Long Sound Velocity (m/s)	Density (kg/m^3^)	Acoustic Impedance ($\times 10^{6}$ kg/(m^2^·s))
Epo-Tek 301/Zirconia	Matching layer	Scheme I	1:1.2	2435	1970	4.79
		Scheme II	1:1.6	2495	2272	5.67
Epo-Tek 301/Tungsten	Backing layer		1:5.5	1589	5320	8.45

**Table 3:** Simulation results of the designed TOFD ultrasonic transducers with scheme I and scheme II.

	*f*_c_ (MHz)	*BW* @ −6 dB (%)	Pulse Length @ −20 dB (μs)	Pk Ampl (dB, re 1 V/V)
Scheme I	7.07	124.7%	0.21	−49.97
Scheme II	6.34	104.8%	0.24	−49.56

### 3.2. Fabrication

**Figure 4:**
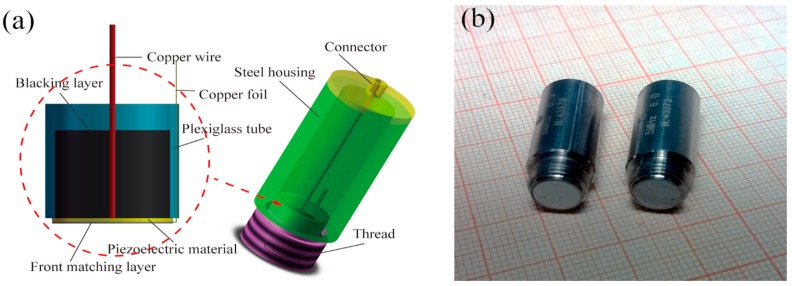
(**a**) Schematic diagram of the designed TOFD ultrasonic transducer; (**b**) Photograph of the fabricated TOFD ultrasonic transducer.

Figure 4a shows the schematic diagram of the designed TOFD ultrasonic transducer with an active element size of 6.0 mm. The major components were the piezoelectric material, matching layer and backing layer. Firstly, considering that the matching layer is very thin, only around 100 µm, the bottom electrode of the active element was led out using a 50 µm thickness copper foil, and then copper wires were soldered to the top electrode using Electrically Conductive Adhesive (E-Solder^®^3022, Von Roll Isola Inc., New Haven, CT, USA). Secondly, the active element was bonded to the plexiglass tube using an Araldite GY2251/HY956 epoxy adhesive, and then the backing layer, matching layer were poured on the surface of active element, respectively. After the layers were cured, the matching layer was carefully lapped to the designed thicknesses, and the waveforms and frequency spectra were monitored at the same time. Finally, the transducer was packed into the stainless steel housing for protection and reduction of electrical noise from surrounding electromagnetic waves. Figure 4b shows a photograph of the fabricated TOFD ultrasonic transducer based on PMNT/epoxy 1–3 composite.

### 3.3. Transducer Performances

The performance of the TOFD ultrasonic transducers was characterized using a conventional pulse-echo response method, and the transmitted pulse was reflected back from a backwall of 12.7 mm polystyrene. The ultrasonic transducer was excited by an electrical impulse energy of 2  μ J with a reputation rate of 1 kHz and a damping factor of 50  Ω using an ultrasonic pulser-receiver (5073PR, Olympus NDT Inc, Waltham, MA, USA). The pulse-echo response in time domain was monitored and captured by an oscilloscope (DSOX4022A, Agilent). The frequency spectrum of echo was obtained by applying FFT, using a function built-in to the oscilloscope. The center frequency (*f*_c_) and −6 dB bandwidth (BW) of the transducers were determined from the measured frequency spectrum:

(6) $$f_{c}=\frac{f_{1}+f_{2}}{2}$$

(7) $$BW=\frac{f_{2}-f_{1}}{f_{c}}\times 100\%$$

where *f*_1_ and *f*_2_ are the lower and upper −6 dB frequencies, respectively.

The relative pulse-echo sensitivity (*S*_rel_), which is the ratio of the output power *P*_o_ of an ultrasonic transducer to the input power *P*_i_ delivered to the ultrasonic transducer from a driving source. By assuming that the input load resistance *R*_i_ and output load resistance *R*_o_ are equal, the *S*_rel_ can be simplified to the following equation:

(8) $$S_{rel}=10\text{log}\left(\frac{P_{0}}{P_{i}}\right)=10\text{log}\left(\frac{V_{0}^{2}/R_{0}}{V_{i}^{2}/R_{i}}\right)=20\text{log}\left(\frac{V_{0}}{V_{i}}\right)$$

The transducer was connected to a function generator (HP8116A, Fremont, CA, USA) which was used to generate a tone burst of 20-cycle sine wave at *f*_c_. The echo’s peak amplitude *V*_o_ was measured by the oscilloscope with 1 MΩ coupling, and the amplitude of the driving signal *V*_i_ was then measured with 50 Ω coupling.

Electrical impedance provides information on the electrical characteristics of a transducer and how it loads a pulser, which was measured in water at the center frequency by an Agilent impedance analyzer 4294A (Agilent Technologies), and the results are given in Table 4.

**Figure 5:**
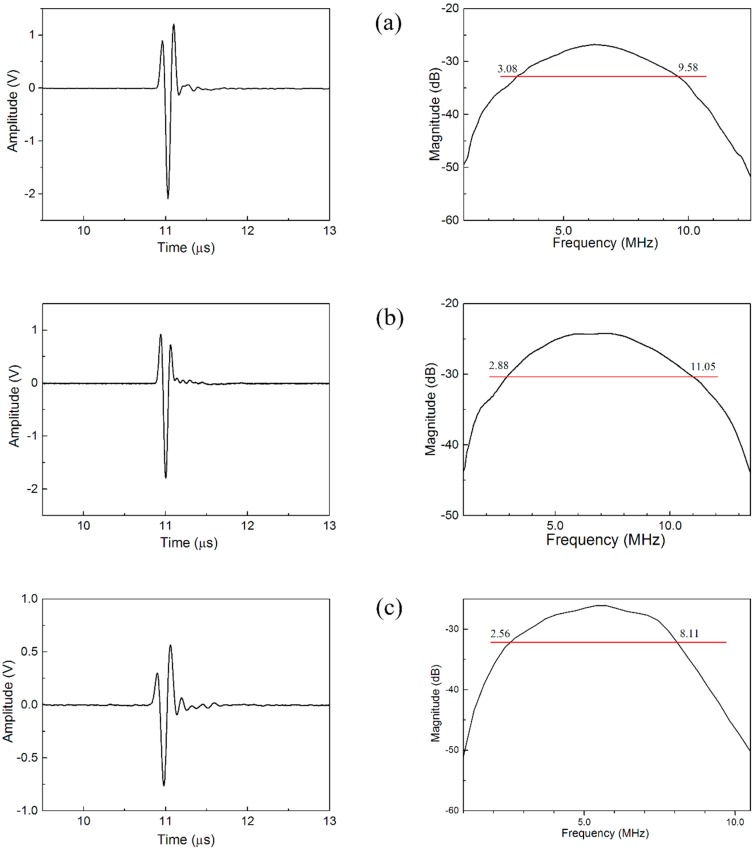
Comparison of the waveforms and frequency spectra of (**a**) PMNT/epoxy 1–3 composite TOFD ultrasonic transducers with scheme I; (**b**) PMNT/epoxy 1–3 composite TOFD ultrasonic transducers with scheme II, and (**c**) PZT/epoxy 1–3 composite TOFD ultrasonic transducers.

**Table 4:** The actual parameters of the TOFD ultrasonic transducers.

	*f*_c_ (MHz)	*BW* @ −6 dB (%)	Pulse Length @ −20 dB (μs)	Relative Pulse-Echo Sensitivity (dB)	Electrical Impedance @ *f*_c_ (Ω)
Scheme I	6.97	117.3	0.21	−24.08	38.7
Scheme II	6.33	102.7	0.23	−21.93	46.2
PZT/epoxy 1–3 composite	5.34	103.9	0.27	−27.96	56.2

Figure 5 shows the waveforms and frequency spectra of the TOFD ultrasonic transducers, including (a) PMNT/epoxy 1–3 composite TOFD ultrasonic transducers with scheme I; (b) PMNT/epoxy 1–3 composite TOFD ultrasonic transducers with scheme II; and (c) PZT/epoxy 1–3 composite TOFD ultrasonic transducer (Changzhou Changchao Testing Equipment Co., Ltd., Changzhou, China). As can be seen clearly from Figure 5, both of the PMNT/epoxy 1–3 composite TOFD ultrasonic transducers have higher amplitudes of pulse-echo and larger bandwidth compared with the PZT/epoxy 1–3 composite TOFD ultrasonic transducer. The actual parameters of these transducers were calculated and listed in Table 4, agreeing well with the simulation results in Table 3. Among two different kinds of TOFD transducer based on PMNT/epoxy 1–3 composite, one exhibits higher detectivity with the relative pulse-echo sensitivity and −6 dB relative bandwidth of −21.93 dB and 102.7% respectively, while another exhibits broader bandwidth with the relative pulse-echo sensitivity and −6 dB relative bandwidth of −24.08 dB and 117.3% respectively. In addition, the TOFD ultrasonic transducers based on PMNT/epoxy 1–3 composite have good damping effect and relatively short waveform duration, being very beneficial to high-resolution NDT applications.

## 4. Conclusions

5–6 MHz PMNT/epoxy 1–3 composites with a volume fraction of 54.8% were prepared by a modified fill-and-dice method. An ultrahigh thickness electromechanical coupling coefficient (85.7%), larger piezoelectric coefficient (1209 pC/N), and relatively lower acoustic impedance (1.82 $\;\times 10^{7}\;$ kg/(m^2^·s)) have been measured in the prepared composites. Based on the high-performance piezoelectric composites, broadband and highly sensitive TOFD ultrasonic transducers were fabricated. In the detection on a backwall of 12.7 mm polystyrene, both of them exhibit considerably improved performance over the PZT/epoxy 1–3 composite TOFD ultrasonic transducer. These results demonstrate that the PMNT/epoxy 1–3 composite is a promising candidate to be used in the ultrasonic transducers for NDT applications.

## References

[1] Kim K.B., Hsu D.K., Ahn B., Kim Y.G., Barnard D.J. (2010). Fabrication and comparison of PMN-PT single crystal, PZT and PZT-based 1–3 composite ultrasonic transducers for NDE applications. Ultrasonics.

[2] Robertson D., Hayward G., Gachagan A., Murray V. (2006). Comparison of the performance of PMN-PT single-crystal and ceramic composite arrays for NDE applications. Insight.

[3] Peng J., Luo H.S., He T.H., Xu H.Q., Lin D. (2005). Elastic, dielectric, and piezoelectric characterization of 0.70Pb(Mg_1/3_Nb_2/3_)O_3_-0.30PbTiO_3_ single crystals. Mater. Lett..

[4] Bokov A.A., Ye Z.G. (2002). Ferroelectric properties of monoclinic Pb(Mg_1/3_Nb_2/3_)O_3_-PbTiO_3_ crystals. Phys. Rev. B.

[5] Luo H.S., Xu G.S., Xu H.Q., Wang P.C., Yin Z.W. (2000). Compositional homogeneity and electrical properties of lead magnesium niobate titanate single crystals grown by a modified bridgman technique. Jpn. J. Appl. Phys. Part 1-Reg. Pap. Short Notes Rev. Pap..

[6] Park S.E., Shrout T.R. (1997). Characteristics of relaxor-based piezoelectric single crystals for ultrasonic transducers. IEEE Trans. Ultrason. Ferroelectr. Freq. Control.

[7] Zhou D., Cheung K.F., Lam K.H., Chen Y., Chiu Y.C., Dai J., Chan H.L.W., Luo H. (2011). Broad-band and high-temperature ultrasonic transducer fabricated using a Pb(In_1/2_Nb_1/2_)O_3_-Pb(Mg_1/3_Nb_2/3_)O_3_-PbTiO_3_ single crystal/epoxy 1–3 composite. Rev. Sci. Instrum..

[8] Chen Y., Lam K.-H., Zhou D., Yue Q., Yu Y., Wu J., Qiu W., Sun L., Zhang C., Luo H. (2014). High performance relaxor-based ferroelectric single crystals for ultrasonic transducer applications. Sensors.

[9] Wang W., Or S.W., Yue Q., Zhang Y., Jiao J., Ren B., Lin D., Leung C.M., Zhao X., Luo H. (2013). Cylindrically shaped ultrasonic linear array fabricated using pimnt/epoxy 1–3 piezoelectric composite. Sens. Actuators A Phys..

[10] Wang W., Or S.W., Yue Q., Zhang Y., Jiao J., Leung C.M., Zhao X., Luo H. (2013). Ternary piezoelectric single-crystal pimnt based 2-2 composite for ultrasonic transducer applications. Sens. Actuators A Phys..

[11] Zhang Y., Zhao X., Wang W., Ren B., Liu D.A., Luo H. (2011). Fabrication of PIMNT/epoxy 1–3 composites and ultrasonic transducer for nondestructive evaluation. IEEE Trans. Ultrason. Ferroelectr. Freq. Control.

[12] Zhang Y., Wang S., Liu D.A., Zhang Q., Wang W., Ren B., Zhao X., Luo H. (2011). Fabrication of angle beam two-element ultrasonic transducers with PMN-PT single crystal and PMN-PTt/epoxy 1–3 composite for NDE applications. Sens. Actuators A Phys..

[13] Zhang Y., Li X., Liu D.A., Zhang Q., Wang W., Ren B., Lin D., Zhao X., Luo H. (2011). The compositional segregation, phase structure and properties of Pb(In_1/2_Nb_1/2_)O_3_-Pb(Mg_1/3_Nb_2/3_)O_3_-PbTiO_3_ single crystal. J. Cryst. Growth.

[14] Wang X., Zhang H., Lin D., Wang S., Zhao X., Chen J., Deng H., Li X., Xu H., Luo H. (2014). An effective growth method to improve the homogeneity of relaxor ferroelectric single crystal Pb(In_1/2_Nb_1/2_)O_3_-Pb(Mg_1/3_Nb_2/3_)O_3_-PbTiO_3_. Cryst. Res. Technol..

[15] Krimholtz R., Leedom D.A., Matthaei G.L. (1970). New equivalent circuits for elementary piezoelectric transducers. Electron. Lett..

[16] Desilets C.S., Fraser J.D., Kino G.S. (1978). The design of efficient broad-band piezoelectric transducers. IEEE Trans. Sonics Ultrason..

